# Perinatal Stress Programs Sex Differences in the Behavioral and Molecular Chronobiological Profile of Rats Maintained Under a 12-h Light-Dark Cycle

**DOI:** 10.3389/fnmol.2019.00089

**Published:** 2019-05-01

**Authors:** Sara Morley-Fletcher, Jerome Mairesse, Gilles Van Camp, Marie-Line Reynaert, Eleonora Gatta, Jordan Marrocco, Hammou Bouwalerh, Ferdinando Nicoletti, Stefania Maccari

**Affiliations:** ^1^UMR 8576, Unité de Glycobiologie Structurale et Fonctionnelle, Campus Cité Scientifique, CNRS, University of Lille, Lille, France; ^2^University Lille – CNRS-UMR 8576, International Associated Laboratory (LIA) “Prenatal Stress and Neurodegenerative Diseases,” Sapienza University of Rome – IRCCS Neuromed, Rome, Italy; ^3^Division of Neonatology, Department of Pediatrics, University of Geneva, Geneva, Switzerland; ^4^Department of Psychiatry, College of Medicine, Psychiatric Institute, Center for Alcohol Research in Epigenetics, University of Illinois at Chicago, Chicago, IL, United States; ^5^Laboratory of Neuroendocrinology, The Rockefeller University, New York, NY, United States; ^6^Department of Physiology and Pharmacology “V. Erspamer,” Sapienza University of Rome, Rome, Italy; ^7^Istituto di Ricovero e Cura a Carattere Scientifico (IRCCS), NEUROMED, Pozzilli, Italy; ^8^Department of Science and Medical – Surgical Biotechnology, Sapienza University of Rome, Rome, Italy

**Keywords:** predictive adaptation, reactive adaptation, circadian rhythms, locomotor activity, chronobiological stressor, mRNA expression

## Abstract

Stress and the circadian systems play a major role in an organism’s adaptation to environmental changes. The adaptive value of the stress system is reactive while that of the circadian system is predictive. Dysfunctions in these two systems may account for many clinically relevant disorders. Despite the evidence that interindividual differences in stress sensitivity and in the functioning of the circadian system are related, there is limited integrated research on these topics. Moreover, sex differences in these systems are poorly investigated. We used the perinatal stress (PRS) rat model, a well-characterized model of maladaptive programming of reactive and predictive adaptation, to monitor the running wheel behavior in male and female adult PRS rats, under a normal light/dark cycle as well as in response to a chronobiological stressor (6-h phase advance/shift). We then analyzed across different time points the expression of genes involved in circadian clocks, stress response, signaling, and glucose metabolism regulation in the suprachiasmatic nucleus (SCN). In the unstressed control group, we found a sex-specific profile that was either enhanced or inverted by PRS. Also, PRS disrupted circadian wheel-running behavior by inducing a phase advance in the activity of males and hypoactivity in females and increased vulnerability to chronobiological stress in both sexes. We also observed oscillations of several genes in the SCN of the unstressed group in both sexes. PRS affected males to greater extent than females, with PRS males displaying a pattern similar to unstressed females. Altogether, our findings provide evidence for a specific profile of dysmasculinization induced by PRS at the behavioral and molecular level, thus advocating the necessity to include sex as a biological variable to study the set-up of circadian system in animal models.

## Introduction

The optimal functioning of an organism is based on its ability to adapt to changes in the environment. The adaptation can be reactive to unexpected environmental changes, or predictive, as it prepares the organism to anticipate the daily changes in the environment (Moore-Ede, 1986). The glucocorticoid stress response is an essential mediator of allostatic processes (McEwen, 1998), which are engaged to allow the organism to find a new equilibrium in response to the specific stressor. This stress response is adaptive when it is transitory and shuts off quickly with the help of feedback loops (McEwen and Stellar, 1993; Maccari et al., 2003, 2017). However, in the long run, prolonged and/or uncontrollable sources of stress lead to a loss of organism’s resistance and entering a phase of exhaustion that might account for many clinically relevant disorders at the behavioral, endocrine, metabolic, cardiovascular, and immune level (de Kloet, 1999; Russell et al., 2018). Glucocorticoids also have a circadian rhythm, and the circadian system is another major physiological system involved in the adaptation of the organism to environmental changes. In contrast to the stress system, whose adaptive value is reactive, the circadian system has a predictive adaptive role (Turek, 2016). As with the stress response, disruption of circadian rhythms leads to clinically relevant disorders including obesity, cardiovascular disease, inflammation, and cognitive impairments (Wirz-Justice and Van den Hoofdakker, 1999; Bunney and Potkin, 2008; Gerstner and Yin, 2010; Wulff et al., 2010; Medic et al., 2017). Circadian rhythms are under control of an internal clock present in nearly every cell, with a core mechanism governed by a genetic network. These clocks are organized hierarchically, with a central pacemaker located in the suprachiasmatic nuclei (SCN) of the hypothalamus at the top, that receive photic cues and in turn coordinate local circadian clocks in the periphery (Moore and Eichler, 1972; Klein et al., 1991; Buijs and Kalsbeek, 2001; Cirelli, 2009). Glucocorticoid hormones and body temperature, among other signals, act as internal systemic synchronizers to entrain peripheral clocks through the SCN (Dibner et al., 2010).

Stress-induced pathologies are often characterized by alterations in circadian rhythms (Weibel et al., 2002; Spiga et al., 2014; Liyanarachchi et al., 2017; Nicolaides et al., 2017), and the glucocorticoid stress response and many clock-controlled processes display sexual dimorphism (for a review, see Darnaudéry and Maccari, 2008; Mong et al., 2011; Heck and Handa, 2018). The corticosterone stress response is markedly greater in adult female rodents than in males (Heck and Handa, 2018), and in both rodents and humans the circadian rhythm of glucocorticoids secretion has higher amplitude in females (Mong et al., 2011; Vestergaard et al., 2017, respectively). In humans, women also display higher rates of sleep disorders than men and a differential ability to adapt to changing shift-work schedules and jet lag (Bailey and Silver, 2014). A sexual dimorphism and first demonstration of gonad-dependence of circadian rhythm concerns circadian locomotor activity in rodents (Albers et al., 1981; Wollnik and Turek, 1988). Gonadal steroids in the SCN seem to act through different mechanisms in males and females. Indeed, androgen receptors are densely located in the core SCN and therefore act on the SCN via a direct pathway, unlike the estrogen receptors that are located mostly extra-SCN and act on SCN afferents (Mong et al., 2011). Nevertheless, the number of studies that associate reactive and predictive adaptations is limited, with the females being underrepresented (Zucker and Beery, 2010; Beery and Zucker, 2011; Kiryanova et al., 2017; McCarthy et al., 2017).

An individual’s capabilities to cope with environmental challenges are shaped during development by the interplay of genetic and epigenetic determinants (McEwen, 2016). Exposure to stressful events in early life strongly programs an individual’s phenotype and adaptive capabilities by modifying reactive (Weaver et al., 2004; Seckl, 2008; Brunton et al., 2011; Maccari et al., 2017) as well as predictive adaptation (Van Reeth et al., 2000; Maccari and Morley-Fletcher, 2007). A well-characterized animal model of early programming of stress response is the PRS model in the rat (Maccari et al., 1995; for a review see Maccari et al., 2017), in which exposure to gestational stress and altered maternal behavior programs a life-long disruption in the reactive adaptation such as a hyperactive response to stress and a defective feedback of the hypothalamus-pituitary-adrenal (HPA) axis (Vallée et al., 1999; Gatta et al., 2018) together with long-lasting modifications in stress/anti-stress gene expression balance in the hippocampus (Mairesse et al., 2015; Gatta et al., 2018). The changes induced by PRS in predictive adaptation include a profound alteration of the sleep-wake cycle architecture together with increased sleep fragmentation when the animals reach adulthood (Dugovic et al., 1999; Mairesse et al., 2013) and alterations in the rhythm of plasma corticosterone secretion (Koehl et al., 1999). Nevertheless, there are no studies on changes in circadian rhythms at the molecular level in the PRS model.

Perinatal stress induces sex-specific effects on reactive and predictive adaptations as well as on sexual maturation induced by gonadal hormones (Reynaert et al., 2016). Indeed, PRS reduces the ratio of testosterone/dihydrotestosterone in males and estradiol levels in females. Such an effect is causally related to sex-dependent alterations in gene expression in the hypothalamus and in the hedonic sensitivity to palatable food (Reynaert et al., 2016). However, studies have generally been conducted in PRS males and have focused on reactive adaptation to a greater extent.

The aim of this study was to explore the influence of PRS on the circadian oscillations of gene expression in the SCN and on circadian locomotor behavior, in a sex-dependent manner. Of note, research on transcriptional rhythms has shown that more than half of all genes in the human and rodent genome follow a circadian pattern (Panda et al., 2002; Hughes et al., 2009; Summa and Turek, 2011; Zhang et al., 2014). Thus, we focused on genes belonging to four functional classes, namely the circadian clock, HPA axis stress response regulation, signaling and glucose metabolism in male and female adult PRS rats. We also monitored the running wheel behavior, first under a regular light/dark (L/D) cycle and then after an abrupt 6-h advance light shift (chronobiological stress).

## Materials and Methods

### Ethics Statement

All the experiments performed in this study followed the guideline of the European Communities Council Directive 2010/63/EU. The protocol of perinatal stress, running wheel behavior and sample collection was approved by the Local Committee CEEA-75 (Comité d’Ethique en Experimentation Animale Nord-Pas de Calais, 75).

### Animals and Perinatal Stress Procedure

#### Animals

In total, 48 nulliparous female Sprague-Dawley rats, weighing approximately 250 g, were purchased from a commercial breeder (Harlan, France). Animals were housed at constant temperature (22 ± 2°C) and under a regular 12 h light/dark (L/D) cycle (light on at 8:00 am). A vaginal smear using endocrine serum (NaCl 0.9%) was performed on the morning following mating with an experienced male. The day on which the smear was sperm positive was considered to be embryonic day 0 (E0). After mating, pregnant females were individually housed with *ad libitum* access to food and water at constant temperature (22°C ± 2°C), and under a regular 12 h light/dark cycle (light on at 08:00). On E11, pregnant females were randomly assigned to stress or control groups (*n* = 24 in each group). Control females were left undisturbed, with an exception made for weighing one time per week in order to follow gestation.

#### Perinatal Stress Procedure

The pregnant female group was subjected to a restraint stress procedure according to a standard protocol (Maccari et al., 1995; Morley-Fletcher et al., 2003). From day 11 of pregnancy until delivery, pregnant female rats were subjected to three stress sessions daily (45 min each), during which they were placed in plastic transparent cylinders with a conical extremity and exposed to bright light or were left undisturbed (control dams). Stress sessions were conducted during the light phase (between 09:00 and 15:00) with a minimum interval of 2 h between each stress session. The local ethical committee approved the gestational restraint procedure. Maternal behavior was monitored for 24 h every day during the first seven post-partum days. Constant monitoring was performed with small infrared cameras placed on the animal cage rack where cages containing lactating females were placed. Within each observation period, the behavior of each mother was scored every minute from post-partum day 1 to day 7 (60 observations/h with 2 h of observation per day, 1 h before lights off and 1 h after lights on). The active behavior of the mother (nursing behavior, grooming, licking, and carrying pups) was scored and the data obtained were expressed as percentages with respect to the total number of observations. Since gestational stress induces a reduction of maternal behavior (Gatta et al., 2018), we refer to the whole procedure as PRS having a prenatal and postnatal effect). In the present study, only male and female offspring from dams presenting a stress-reduced maternal behavior (with a cutoff below 40% of maternal care in the PRS group vs. a cutoff above 60% of maternal care in the control group), and from litters of 10–14 rats with a similar number of males and females, were used. Weaning of the offspring occurred at 21 days after birth.

### Experimental Design

Separate sets of rats were used for behavioral studies (*n* = 40, 10 rat/sex/group) and transcriptomic analysis (*n* = 60, *n* = 5/group/time point). Within each litter, we took only two males and two females to minimize any litter effect (Chapman and Stern, 1979). Therefore, there were no more than two sibling within the same time point for the transcriptomic study. Housing conditions were two same-sex animals per cage from each perinatal and experimental group (CONT vs. PRS and behavioral or transcriptomic study) after weaning and until experiments started (at 4 months of age).

### Running Wheel Activity

A first group of rats (*n* = 10/sex/group) was used to analyze the rhythm of circadian activity under a regular 12/12 L/D cycle (08:00–20:00). Rats were housed in light, air tight chambers equipped with continuously operating ventilating fans and single-housed in individual cages equipped with a running wheel that allowed continuous recording of locomotor activity via an on-line computer (Chronobiology kit, Stanford Software System, CA, United States), with light intensity set at 30–40 lux at cage floor level. Each chamber contained 10 single-cages, and males and females were housed separately. During the course of the experiments, food and water were provided *ad libitum*; room temperature (22°C) and humidity (60%) were kept constant. After 10–15 days of adaptation to the running wheels, the rhythm of activity was individually analyzed over 10 consecutive days. The onset of activity was identified with a 5 min resolution and was defined as the first time point at which the mean intensity of activity was above 10% of the maximum and remained above that point for at least 50% of the time during the following 30 min. The reversed procedure was used for the cessation (offset) of activity (first time point below 10% of maximum and activity remained below that point for at least 50% of the time during the following 30 min). The time elapsed between the onset and offset of activity was defined as the total time of nocturnal activity, the peak value of activity and peak hour of activity were directly determined on the actogram for each animal. The mean 24 h integrated activity was determined by adding the mean number of revolutions in the wheel, every 5 min over 10 consecutive days for each animal. The data were then plotted with a 30 min or 5 min resolution; this represented the mean number of wheel revolutions performed by the animals.

### Exposure to a Chronobiological Stressor

At the end of the recording of normal activity, rats (*n* = 10/sex/group) were subjected to an abrupt 6-h advance shift in the L/D cycle. On the day of the shift, lights were turned-off 6 h before the current time, and the new 12 h L/D cycle (light on, 02:00) was maintained thereafter. The time to resynchronize to the new L/D cycle was defined as the number of days for the animal to exhibit a regular activity for at least three consecutive days. The time taken for re-entrainment to the new L/D cycle was individually assessed using the onset of activity criterion determined as previously described. This criterion was defined as the smallest number of days required for the shifted activity onset to occur within 30 min of lights-off and to be stable for 3 days under the new L/D cycle.

### Gene Expression Analysis in the Suprachiasmatic Nucleus (SCN)

A separate set of male and female CONT and PRS rats was used for gene expression analysis in the brain (*n* = 5 per group/time point) at three selected time points (15:00, 19:00 and 02:00) under a regular 12/12 L/D cycle (with light on at 08:00) in order to extract SCN during light phase (15:00), before the onset of dark phase (19:00) and during it (02:00).

The SCN of male and female CONT and PRS rats was rapidly dissected and kept frozen at -80°C. Special attention was taken for tissue collection at 2 am: in order to avoid any influence of light on gene expression, animals were killed under red light before tissue dissection, which did occurr under white light. RNA extraction was performed using the RNeasy Plus mini kit (Qiagen, France) following manufacturer’s instructions. RNA concentration was determined using Nanodrop (ND-1000, Labtech, Germany), and quality verified by RIN (RNA Integrity Number; Bioanalyzer 2100, Agilent Technologies, France). All RNA samples had a quality score above 7.5 RIN. Retrotranscription was performed with the High-Capacity cDNA Reverse Transcription kit (Applied Biosystems, France). Transcript levels were measured by Custom Taqman qRT-PCR (Applied Biosystems, France). The following Taqman real-time PCR probes were obtained from Applied Biosystems:

#### Clock-Related Genes

Clock (Rn00573120_m1), aryl hydrocarbon receptor nuclear translocator-like (Bmal, Arntl, Rn00577590_m1), Cry1 (Rn01503063), period circadian regulator 1 (Per1, Rn01496757_m1), period circadian regulator 2 (Per2, Rn01426757_m1), period circadian regulator 3 (Per3, Rn00709499_m1), nuclear receptor subfamily 1-group D- member 1 (Rev-erbA-α, Nrd1, Rn00595671_m1), melatonin-1A receptor (Mel1AR, Rn01488022_m1), melatonin-1B receptor (Mel1BR, Rn01447987_m1), neuronal PAS domain protein 2 (Npas2, Rn01438223_m1).

#### HPA Axis Stress-Related Genes

Corticotropin releasing hormone receptor 1 (CrhR1, Rn00578611_m1), corticotropin releasing hormone receptor 2 (CrhR2, Rn00575617_m1), glucocorticoid receptor (GR, Nrc1, Rn00565562_m1), mineralocorticoid receptor (MR, Nrc2, Rn00561369_m1), Arginin vasopressin receptor 1a (Avpr1a, Rn00583910_m1), oxytocin receptor (OxtR, Rn00563503_m1).

#### Signaling-Related Genes

Metabotropic glutamate receptor 2 (mGluR2, Grm2, Rn01447672_m1), metabotropic glutamate receptor 3 (mGluR3, Grm3, Rn01755352_m1), metabotropic glutamate receptor 5 (mGluR5, Grm5, Rn00566628_m1), 5-hydroxytryptamine receptor 2C (Htr2c, Rn00562748_m1), mechanistic target of rapamycin (mTor, Rn00693900_m1), v-akt murine thymoma viral oncogene homolog 1 (Akt1, Rn00583646_m1), disrupted in schizophrenia 1 (Disc1, Rn00598264_m1), dickkopf WNT signaling pathway inhibitor 1 (Dkk1, Rn01501537_m1), early-growth transcription factor 1 (Ngf1A, Egr1, Rn00561139_m1), erb-b2 receptor tyrosine kinase 2 (Erbb2, Rn00566561_m1), erb-b2 receptor tyrosine kinase 3 (Erbb3, Rn00568107_m1), erb-b2 receptor tyrosine kinase 4 (Erbb4, Rn00572447_m1), solute carrier family 17-member 7, vesicle bound sodium-dependent inorganic phosphate cotransporter (vGlut1, Slc17a7, Rn01462431_m1), solute carrier family 1 member 3, glial high affinity glutamate transporter (Glast, Slc1a3, Rn00570130_m1).

#### Glucose Metabolism-Related Genes

Glycogen synthase kinase 3 beta (Gsk3ß, (Rn00583429_m1), insulin-like growth factor 1 (Igf1, Rn00710306_m1), insulin-like growth factor 1 receptor (Igf1R, Rn00583837_m1), insulin receptor (InsR, Rn00690703_m1), phosphoinositide-3-kinase regulatory subunit 1 (Pik3R1, Rn00564547_m1), solute carrier family 2, facilitated glucose transporter, member 1 (Glut1, Slc2a1, Rn01417099_m1), solute carrier family 2, facilitated glucose transporter, member 3 (Glut3, Slc2a3, Rn00567331_m1), solute carrier family 2, facilitated glucose transporter, member 4 (Glut4, Slc2a4, Rn00562597_m1), O-GlcNAcase (Oga, Mgea5, Rn00590870_m1), O-GlcNAc-transferase (Ogt, Rn00820779_m1), glutamine fructose-6-phosphate transaminase 1 (Gfpt1, Rn01765495_m1) and glutamine fructose-6-phosphate transaminase 2 (Gfpt2, Rn01456720_m1).

Transcript levels were normalized based on endogenous glyceraldehyde-3-phosphate dehydrogenase (Gapdh, Rn01462662_g1) and tyrosine 3-monooxygenase/tryptophan 5-monooxygenase activation protein (Ywhaz, Rn00755072_m1) expression.

Assay validity was assessed by using appropriate negative controls, in which cDNA was omitted. These negative controls were not read by the software. Acquisition of data (threshold cycle, Ct) was performed by StepOnePlus^TM^ software. A ΔCt (Ct of the considered gene – mean Ct (Gapdh and Ywhaz genes) was calculated.

### Statistical Analysis

All data were analyzed using two-way Analysis Of Variance ANOVA, with group (CONT vs. PRS) and sex (Males vs. Females) as independent variables, and time (hours) as repeated measure. Fisher’s LSD test was used to analyze the differences. Significance was set at a *p*-value of 0.05. In mRNA analysis, in order to subdivide data accounting for significant lower-order effects when F was larger than, trends with 0.05 < *p* < 0.08 were also considered as significant (Snedecor and Cochran, 1967).

## Results

### PRS Induces a Sex-Dependent Disruption of the Circadian Rhythm of Running Wheel Activity

**FIGURE 1:**
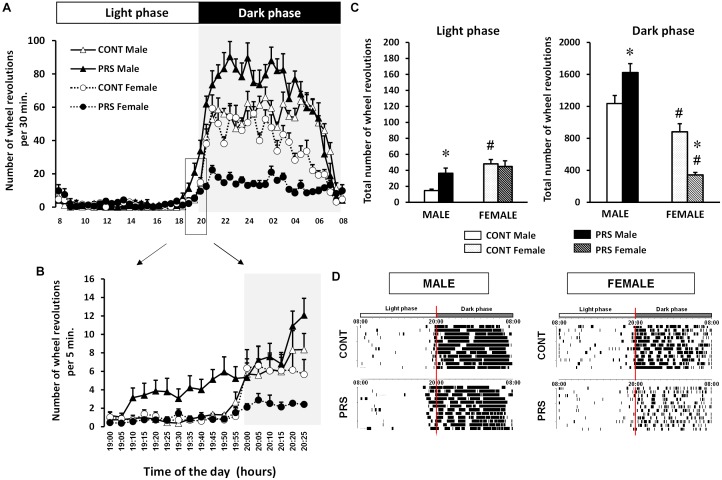
Circadian locomotor activity in a wheel running paradigm in PRS and CONT animals of both sexes. **(A)** The 24 h representation of the mean number of wheel revolutions calculated over 10 consecutive days and plotted with a 30 min resolution, obtained from adult PRS and CONT rats of both sexes showing hyperactivity in PRS males and hypoactivity in PRS females. **(B)** Details of the light switch off period with data plotted with a 5 min resolution allowing a clear visualization of the advance in activity phase in male PRS animals. **(C)** Total number of wheel revolutions over 10 consecutive days during the period of inactivity (light phase) and activity (dark phase) showing the sex effect in the CONT group with females being more active in the light and less active in the dark phase. This effect was observed in the PRS group only in the dark phase, with females being hypoactive, and PRS males being always hyperactive. **(D)** Representative activity records of the circadian running wheel activity of male and female PRS and CONT rats over 10 consecutive days, where the phase advance in PRS males and fragmented activity in both PRS males and females is clearly observed. The first L/D cycle is shown at the top of each panel. Each horizontal line represents 24 h of the animal’s life composed from the vertical bar, each representing a wheel revolution. Values are expressed as means ± S.E.M. *n* = 10 rats per group. ^∗^*p* < 0.05 PRS *vs.* CONT within the same sex group; ^#^*p* < 0.05 females *vs*. males within the CONT or PRS group.

The circadian rhythms of running wheel activity under a regular 12/12 LD cycle were individually analyzed in male and female CONT unstressed and PRS rats over 10 consecutive days of continuous registration (Figure 1A). PRS induced changes in the circadian rhythm (*Group x Sex x Time effect, F*_(1,47)_ = 13.241, *p* < 0.05) with PRS males anticipating the light being switched off by starting the wheel running activity at 19:10 (Figure 1B); in contrast, all the other groups started around 20:00 (which is the precise time of light switch off). PRS females presented reduced number of wheel revolutions with respect to all other groups during the dark phase (Figure 1A,C). Indeed, there was a clear-cut effect of sex on the effect of PRS on the levels of activity defined by the number of wheel revolutions (Figure 1C); first, during the period of lower activity (light phase), both CONT and PRS females were more active than males. During the light phase, PRS increased activity in males, which reached levels of CONT females (*Group x Sex effect, F*_(1,36)_ = 7.68, *p* < 0.01). More interestingly, during the period of activity (dark phase), male PRS rats were more active than male CONT rats, in contrast, female PRS rats were less active than CONT females (*Group x Sex effect, F*_(1,36)_ = 39.48, *p* < 0.01). In addition, during the dark phase, CONT female rats were less active than CONT male rats. Overall, PRS males presented a phase advance and increased wheel activity, while PRS females were hypoactive and presented a more fragmented profile of activity than did CONT females (Figure 1D).

### PRS Increases Vulnerability to Chronobiological Stress

**FIGURE 2:**
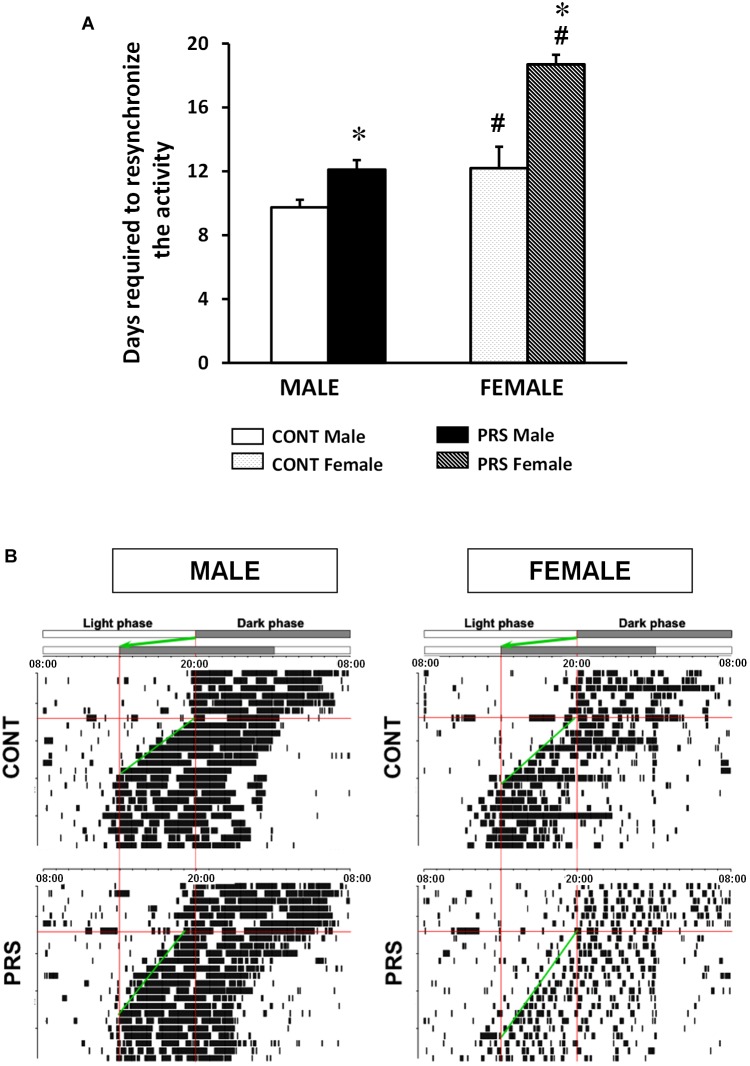
Locomotor response to a chronobiological stressor in PRS and CONT animals of both sexes. **(A)** Circadian running wheel activity from CONT-unstressed and PRS rats of both sexes that were subjected to abrupt 6-h phase advance of the L/D cycle. PRS increased the time taken for resynchronization to the new L/D cycle in both sexes, showing increased sensitivity to the chronobiological stressor in PRS animals. Moreover, this occurred to a larger extent in females than in male PRS rats, with 7 days required by PRS females and 3 days by PRS males. **(B)** Representative activity records of the circadian running wheel activity on day 7. The first L/D cycle and the new L/D cycle, after a 6-h advance in the light period are shown at the top of each panel. Values are expressed as mean ± S.E.M. *n* = 10 rats per group. ^∗^*p* < 0.05 PRS *vs.* CONT within the same sex group; ^#^*p* < 0.05 females *vs.* males within the CONT or PRS group.

We measured the number of days needed for the circadian rhythm of locomotor activity to become resynchronized after an abrupt 6-h advance shift in the L/D cycle (Figure 2A). Both PRS and sex influenced the time necessary for resynchronization (ANOVA, *Group x Sex effect, F*_(1,36)_ = 6.316, *p* < 0.05). In the CONT group, females required a longer time to resynchronize to the new light shift than the males did (*p* < 0.05). PRS increased the time to become resynchronized to the new L/D cycle in both sexes with PRS males reaching levels of CONT females. Moreover, the PRS effect occurred to a larger extent in female than in males (*p* < 0.05 vs. PRS males). Further, PRS females presented a more fragmented activity than PRS males (Figure 2B).

### Effect of PRS and Sex on Circadian Gene Expression in the SCN

The expression of the 42 genes analyzed in the SCN during discrete time points (15:00, 19:00 and 02:00) is reported in Table 1, with genes such as *Gsk3ß, Igf1, mGluR2, mGluR3, Dkk1*, and *Glast* that were only influenced by PRS or sex (Supplementary Figure S1) and the other genes that followed a circadian rhythm in a sex-dependent manner and were also affected by PRS. Seven genes, did not show changes associated with PRS, sex, or time (*CrhR2, mGluR5, Htr2c, Erbb2, Erbb3, Erbb4, Gfpt1, Gfpt2*).

**Table 1:** Effect of perinatal stress and sex on the temporal gene expression in the SCN.

		Males						Females						
			
		CONT			PRS			CONT			PRS			
					
Gene name	Statistical effect	15	19	02	15	19	02	15	19	02	15	19	02	Taqman ID (RnX_m1)
Clock	GST	1.00 ± 0.01	0.91 ± 0.03	1.16 ± 0.11	0.89 ± 0.01	0.94 ± 0.01	1.06 ± 0.06	0.98 ± 0.03	0.93 ± 0.05	**0.90^#^ ± 0.04**	0.93 ± 0.02	0.91 ± 0.05	**1.05^∗^ ± 0.04**	00573120
Bmal	GST	1.00 ± 0.04	0.79 ± 0.06	1.12 ± 0.13	0.97 ± 0.04	0.81 ± 0.04	1.13 ± 0.11	1.11 ± 0.03	**1.07^#^ ± 0.09**	0.96 ± 0.06	**0.89^∗^ ± 0.03**	0.88 ± 0.05	**1.28^∗^ ± 0.13**	00577590
Oga	GST	1.00 ± 0.03	0.95 ± 0.07	1.08 ± 0.11	1.05 ± 0.04	0.90 ± 0.02	1.00 ± 0.05	1.13 ± 0.04	1.01 ± 0.07	**0.89^#^ ± 0.04**	**0.94^∗^ ± 0.01**	0.96 ± 0.09	**1.09^∗^ ± 0.07**	00590870
Ngf1A	GST	1.00 ± 0.24	0.56 ± 0.08	0.52 ± 0.14	**0.66^∗^ ± 0.12**	0.73 ± 0.05	**0.91^∗^ ± 0.08**	0.76 ± 0.07	0.61 ± 0.09	0.56 ± 0.09	0.86 ± 0.11	0.80 ± 0.04	**0.62^#^ ± 0.04**	00561138
Rev-erbA-α	GST	1.00 ± 0.06	1.01 ± 0.09	0.55 ± 0.07	1.12 ± 0.07	**0.85^∗^ ± 0.05**	0.61 ± 0.06	1.12 ± 0.02	**0.85^#^ ± 0.04**	0.58 ± 0.02	1.04 ± 0.03	0.95 ± 0.08	0.52 ± 0.02	00595671
Mel1AR	GST	1.00 ± 0.37	0.77 ± 0.24	0.47 ± 0.09	1.38 ± 0.46	**1.74^∗^ ± 0.37**	0.59 ± 0.22	1.09 ± 0.22	**2.19^#^ ± 0.39**	0.68 ± 0.21	0.87 ± 0.28	**0.90^∗#^ ± 0.26**	0.49 ± 0.02	01488022
CrhR1	GS/T	1.00 ± 0.06	0.91 ± 0.03	1.08 ± 0.06	**0.80^∗^ ± 0.03**	0.81 ± 0.04	**0.94^∗^ ± 0.09**	**0.84^#^ ± 0.02**	**0.75^#^ ± 0.03**	**0.86^#^ ± 0.04**	0.84 ± 0.03	0.83 ± 0.04	0.85 ± 0.03	00578611
MR	GS/T	1.00 ± 0.02	0.94 ± 0.03	1.07 ± 0.07	**0.86^∗^** ± 0.01	0.90 ± 0.02	0.99 ± 0.07	0.97 ± 0.03	0.95 ± 0.04	0.96 ± 0.01	0.96 ± 0.02	0.95 ± 0.08	1.01 ± 0.04	00561369
Mtor	GS/T	1.00 ± 0.01	0.91 ± 0.03	1.00 ± 0.04	**0.87^∗^ ± 0.02**	0.92 ± 0.01	0.99 ± 0.05	0.93 ± 0.02	0.86 ± 0.03	**0.92^#^ ± 0.02**	0.95 ± 0.01	0.91 ± 0.03	0.92 ± 0.04	00693900
Glut1	GS/T	1.00 ± 0.06	1.13 ± 0.09	1.27 ± 0.17	0.84 ± 0.03	0.99 ± 0.04	1.15 ± 0.09	0.80 ± 0.02	**0.88^#^ ± 0.03**	**0.93^#^ ± 0.02**	0.84 ± 0.03	1.08 ± 0.17	0.93 ± 0.03	01417099
vGlut	GT/ST	1.00 ± 0.28	0.76 ± 0.11	2.40 ± 0.38	1.23 ± 0.50	0.94 ± 0.19	**3.62^∗^ ± 0.56**	1.66 ± 0.45	0.80 ± 0.13	**0.73^#^ ± 0.13**	1.77 ± 0.14	1.29 ± 0.17	**2.02^∗#^ ± 0.23**	01462431
Mel1BR	ST	1.00 ± 0.16	1.08 ± 0.22	1.39 ± 0.48	1.32 ± 0.14	1.03 ± 0.15	1.30 ± 0.22	1.14 ± 0.18	1.14 ± 0.17	**0.82^#^ ± 0.13**	0.89 ± 0.09	1.18 ± 0.10	**0.73^#^ ± 0.09**	01447987
Npas2	ST	1.00 ± 0.02	0.83 ± 0.04	1.23 ± 0.13	0.88 ± 0.03	0.90 ± 0.04	1.23 ± 0.04	0.90 ± 0.01	0.86 ± 0.05	**0.97^#^ ± 0.03**	0.89 ± 0.01	0.86 ± 0.06	**1.04^#^ ± 0.06**	01438223
Disc1	ST	1.00 ± 0.09	0.95 ± 0.03	1.45 ± 0.25	0.88 ± 0.04	0.96 ± 0.09	1.48 ± 0.17	0.82 ± 0.03	1.01 ± 0.09	**0.98^#^ ± 0.02**	0.91 ± 0.05	1.04 ± 0.12	**0.92^#^ ± 0.09**	00598264
Glut4	ST	1.00 ± 0.15	1.61 ± 0.39	1.92 ± 0.34	1.06 ± 0.10	1.16 ± 0.09	2.15 ± 0.35	1.01 ± 0.08	1.34 ± 0.04	**1.27^#^ ± 0.13**	1.00 ± 0.12	1.31 ± 0.23	**1.08^#^ ± 0.11**	00562597
Igf1R	ST	1.00 ± 0.03	1.04 ± 0.05	1.26 ± 0.13	0.94 ± 0.02	0.95 ± 0.02	1.38 ± 0.17	1.01 ± 0.01	1.11 ± 0.03	1.11 ± 0.03	1.03 ± 0.02	**1.17^#^ ± 0.14**	**1.08^#^ ± 0.03**	00583837
Pik3R1	ST	1.00 ± 0.07	1.17 ± 0.07	1.32 ± 0.09	1.03 ± 0.04	1.11 ± 0.04	1.27 ± 0.07	**1.24^#^ ± 0.05**	1.14 ± 0.05	**1.11^#^ ± 0.03**	1.11 ± 0.05	1.23 ± 0.17	1.21 ± 0.04	00564547
Cry1	S/T	1.00 ± 0.04	1.23 ± 0.07	1.47 ± 0.22	1.00 ± 0.04	1.14 ± 0.07	1.39 ± 0.10	0.90 ± 0.03	1.05 ± 0.06	1.32 ± 0.05	0.89 ± 0.01	1.16 ± 0.10	1.33 ± 0.03	01503063
Per1	S/T	1.00 ± 0.08	1.13 ± 0.12	0.89 ± 0.08	0.85 ± 0.11	1.04 ± 0.05	1.03 ± 0.11	0.84 ± 0.06	0.92 ± 0.05	0.76 ± 0.01	0.86 ± 0.02	1.15 ± 0.16	0.79 ± 0.06	01496757
Avpr1a	S/T	1.00 ± 0.09	1.13 ± 0.10	1.32 ± 0.22	1.04 ± 0.05	1.05 ± 0.04	1.40 ± 0.10	1.14 ± 0.04	1.10 ± 0.02	1.51 ± 0.09	1.18 ± 0.07	1.18 ± 0.11	1.43 ± 0.08	00583910
Akt1	S/T	1.00 ± 0.03	0.95 ± 0.01	1.07 ± 0.04	0.91 ± 0.03	0.92 ± 0.03	1.13 ± 0.13	**0.86^#^ ± 0.03**	0.89 ± 0.03	**0.93^#^ ± 0.02**	0.96 ± 0.01	0.91 ± 0.05	**0.93^#^ ± 0.02**	00583646
nsR	S/T	1.00 ± 0.02	0.90 ± 0.04	1.08 ± 0.12	0.91 ± 0.02	0.88 ± 0.02	0.97 ± 0.07	0.92 ± 0.03	0.85 ± 0.02	0.88 ± 0.02	0.88 ± 0.02	0.90 ± 0.05	0.95 ± 0.03	00690703
Gsk3b	GS	1.00 ± 0.02	1.01 ± 0.03	1.01 ± 0.02	0.95 ± 0.03	0.95 ± 0.04	0.92 ± 0.02	0.96 ± 0.01	0.88 ± 0.04	0.94 ± 0.03	0.93 ± 0.02	0.93 ± 0.06	0.91 ± 0.04	00583429
Igf1	S	1.00 ± 0.13	1.16 ± 0.16	1.08 ± 0.11	1.13 ± 0.08	1.18 ± 0.05	1.28 ± 0.12	1.80 ± 0.46	1.74 ± 0.19	1.59 ± 0.12	1.68 ± 0.25	1.75 ± 0.14	1.91 ± 0.25	00710306
mGlu2	S	1.00 ± 0.15	0.90 ± 0.12	1.04 ± 0.13	1.02 ± 0.07	0.70 ± 0.10	1.19 ± 0.39	0.92 ± 0.09	0.60 ± 0.11	0.52 ± 0.08	0.72 ± 0.10	0.79 ± 0.09	0.68 ± 0.07	01447672
mGlu3	S	1.00 ± 0.07	0.98 ± 0.08	1.11 ± 0.10	0.82 ± 0.04	0.81 ± 0.06	1.20 ± 0.26	0.89 ± 0.05	0.78 ± 0.10	0.82 ± 0.04	0.82 ± 0.04	0.95 ± 0.12	0.89 ± 0.06	01755352
Dkk1	S	1.00 ± 0.12	0.89 ± 0.09	1.46 ± 0.20	1.03 ± 0.11	0.87 ± 0.09	1.63 ± 0.38	1.04 ± 0.15	1.74 ± 0.39	1.17 ± 0.22	1.41 ± 0.19	1.22 ± 0.27	1.56 ± 0.09	01501537
Glast	S	1.00 ± 0.03	1.06 ± 0.03	1.05 ± 0.08	1.02 ± 0.03	0.99 ± 0.08	1.22 ± 0.14	0.98 ± 0.02	0.93 ± 0.03	0.97 ± 0.03	0.98 ± 0.02	0.99 ± 0.03	0.94 ± 0.02	00570130
Per2	T	1.00 ± 0.05	1.43 ± 0.13	1.12 ± 0.11	0.98 ± 0.08	1.27 ± 0.09	1.19 ± 0.11	1.03 ± 0.01	1.22 ± 0.05	1.00 ± 0.04	1.02 ± 0.02	1.38 ± 0.20	0.91 ± 0.05	01427704
Per3	T	1.00 ± 0.02	1.20 ± 0.07	1.09 ± 0.11	0.93 ± 0.05	1.11 ± 0.02	1.04 ± 0.07	0.97 ± 0.02	1.10 ± 0.05	1.07 ± 0.04	0.94 ± 0.01	1.25 ± 0.18	0.96 ± 0.03	00709499
GR	T	1.00 ± 0.03	1.04 ± 0.07	1.29 ± 0.12	0.95 ± 0.04	1.04 ± 0.07	1.16 ± 0.07	1.01 ± 0.05	1.08 ± 0.06	1.09 ± 0.06	0.95 ± 0.02	1.07 ± 0.10	1.04 ± 0.07	00565562
OxtR	T	1.00 ± 0.16	0.97 ± 0.16	0.76 ± 0.12	0.89 ± 0.12	1.23 ± 0.19	0.96 ± 0.20	0.87 ± 0.08	1.05 ± 0.11	0.75 ± 0.03	0.87 ± 0.07	1.22 ± 0.26	1.01 ± 0.11	00563503
Glut3	T	1.00 ± 0.03	0.88 ± 0.02	0.99 ± 0.04	0.87 ± 0.02	0.91 ± 0.03	0.99 ± 0.04	0.95 ± 0.02	0.87 ± 0.04	0.95 ± 0.03	0.93 ± 0.00	0.89 ± 0.03	0.91 ± 0.05	00567331
Ogt	T	1.00 ± 0.06	1.02 ± 0.04	1.24 ± 0.14	1.09 ± 0.06	1.26 ± 0.10	1.31 ± 0.18	1.28 ± 0.05	1.07 ± 0.09	1.17 ± 0.08	1.00 ± 0.03	1.18 ± 0.14	1.26 ± 0.11	00820779
mGlu5	–	1.00 ± 0.08	0.82 ± 0.05	0.88 ± 0.06	0.90 ± 0.06	0.91 ± 0.04	1.10 ± 0.16	0.94 ± 0.04	0.83 ± 0.07	0.88 ± 0.04	0.95 ± 0.04	0.82 ± 0.06	0.84 ± 0.03	00566628
Htr2c	–	1.00 ± 0.06	1.05 ± 0.06	1.10 ± 0.17	1.07 ± 0.09	1.03 ± 0.11	1.00 ± 0.22	0.93 ± 0.06	1.17 ± 0.17	0.92 ± 0.05	0.85 ± 0.01	1.23 ± 0.08	1.00 ± 0.07	00562748
CrhR2	–	1.00 ± 0.12	0.99 ± 0.08	0.89 ± 0.07	0.92 ± 0.11	1.05 ± 0.11	0.86 ± 0.02	1.00 ± 0.11	1.00 ± 0.12	1.00 ± 0.05	1.07 ± 0.07	1.00 ± 0.06	0.94 ± 0.03	00575617
ErbB2	–	1.00 ± 0.09	1.07 ± 0.07	1.00 ± 0.12	1.02 ± 0.03	1.02 ± 0.04	1.12 ± 0.11	0.97 ± 0.06	1.37 ± 0.15	1.07 ± 0.07	0.95 ± 0.07	1.15 ± 0.11	1.04 ± 0.05	00566561
ErbB3	–	1.00 ± 0.07	1.01 ± 0.09	1.27 ± 0.13	0.87 ± 0.05	0.93 ± 0.07	0.96 ± 0.11	1.01 ± 0.06	0.97 ± 0.07	1.09 ± 0.06	0.90 ± 0.04	1.07 ± 0.10	1.01 ± 0.09	00568107
ErbB4	–	1.00 ± 0.03	0.97 ± 0.03	1.12 ± 0.12	0.93 ± 0.03	0.91 ± 0.03	1.07 ± 0.10	1.08 ± 0.06	0.93 ± 0.02	0.92 ± 0.03	0.96 ± 0.02	0.99 ± 0.07	0.98 ± 0.06	00572447
Gfpt1	–	1.00 ± 0.03	0.99 ± 0.04	1.11 ± 0.08	0.96 ± 0.04	0.97 ± 0.03	1.06 ± 0.04	1.01 ± 0.04	0.97 ± 0.02	0.97 ± 0.02	0.99 ± 0.02	1.00 ± 0.06	1.00 ± 0.04	01765495
Gfpt2	–	1.00 ± 0.06	0.97 ± 0.04	1.10 ± 0.09	0.93 ± 0.03	0.90 ± 0.02	1.12 ± 0.11	0.90 ± 0.04	0.93 ± 0.03	0.95 ± 0.02	0.92 ± 0.04	1.00 ± 0.10	0.95 ± 0.06	01456720


*Expression of selected genes in the SCN of unstressed or PRS rats of both sexes was assayed by Custom Taqman qRT-PCR. For each sample, expression levels were normalized to the average levels of unstressed male rats obtained at 15:00 and expressed as Relative Quantitation (RQ). Genes are listed according to the main statistical effect: GST, Group × Stress × Time effect; GS, Group × Sex effect; GT, group × time; ST, sex × time effect; G, S, T for Group, Sex, or Time effect only. Data are means ± S.E.M of five determinations for each group. ^∗^*p* < 0.05 for PRS vs. CONT of the same-sex group; ^#^*p* < 0.05 for females vs. males within the PRS or CONT group. Bolded values are those with greater significance.*

#### Signaling- and Glucose Metabolism -Related Genes

The expression of genes involved in signaling such as *mTor, Akt1, Disc1, Dkk1, mGluR2, mGluR3*, and *Glast* or in glucose metabolism, such as *Igf1, InsR, Gsk3ß* and *Glut1*, displayed a marked effect of both sex and PRS variables or sex only, whereas circadian oscillations were evident for *mTor, Glut1, Akt1, Disc1*, and *InsR*. As shown in Supplementary Figure S1, most of the sex-biased genes were down-regulated in females, with only *Dkk1* and *Igf1* being upregulated. Interestingly, PRS in males downregulated levels of gene expression compared to CONT males, with levels identical to CONT females (*mTor, Gsk3ß, and Glut1*).

#### Clock- and Glucose Metabolism-Related Genes

**FIGURE 3:**
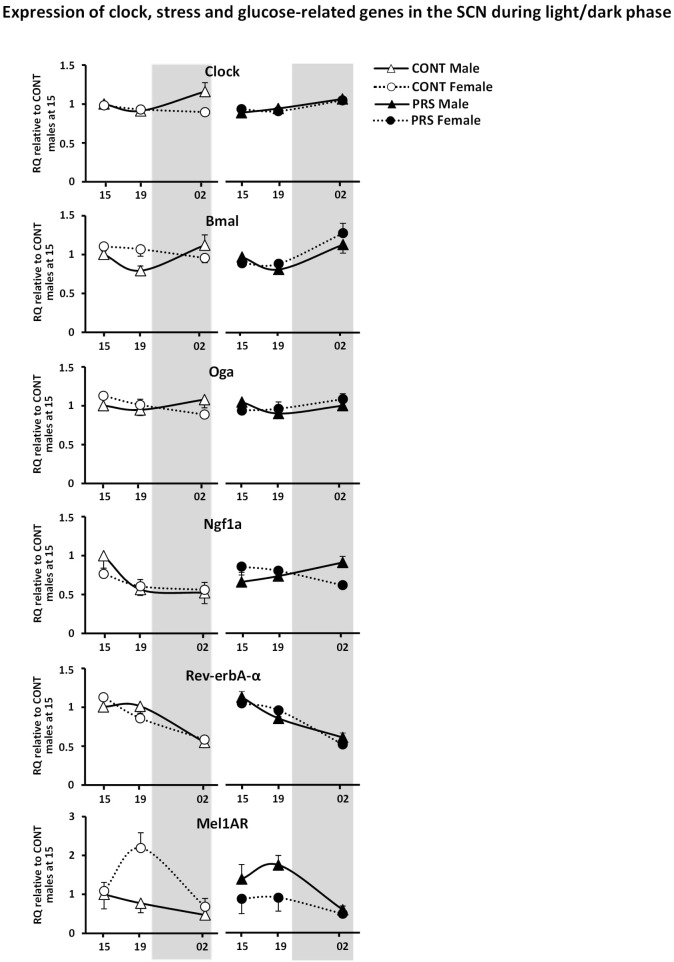
Circadian expression of clock and glucose metabolism-related genes in the SCN of PRS and CONT animals of both sexes. Expression of selected genes in the SCN of CONT or PRS rats of both sexes was assayed by Custom Taqman qRT-PCR. Expression levels of each sample were normalized to the average levels in CONT male rats obtained at 15:00 and expressed as Relative Quantitation (RQ). Effect of PRS on gene expression was observed in both sexes for all the genes considered. PRS induced a dysmasculinization in the profile of expression for *Clock* and *Mel1aR* in males, which showed the same expression profile as CONT females. In *Bmal, Rev-erb-*α and *Mel1aR*, PRS females behaved as CONT males. Data are depicted as mean ± S.E.M of five determinations for each group.

Interestingly, we observed circadian variations in gene expression that were influenced by both PRS and sex, in the genes that were measured and were involved in clock and glucose metabolism regulation, such as *Clock, Bmal, Mel1aR, Rev-erbA-α, Ngf1A*, and *Oga* (Figure 3). Among these genes, sex differences observed in the unstressed group for *Clock* and *Bmal* disappeared in the PRS group. mRNA levels of *Ngf1A* and *Oga* in PRS males showed opposing oscillations when compared to the CONT male group, and this occurred specifically before the onset of the dark phase (19:00) or during it (02:00). Additionally, PRS flipped the sex-specific profile observed in Rev-erbA-α and Mel1aR when compared to CONT, with PRS males showing the same profile that CONT females, and PRS females displaying an expression profile mimicking CONT males (ANOVA, *Group x Sex x Time effect*, Clock, *F*_(2,32)_ = 3.229, *p* = 0.05; Bmal, *F*_(2,32)_ = 3.901, *p* < 0.05; Ngf1A, *F*_(2,32)_ = 3.595, *p* < 0.05; Oga, *F*_(2,32)_ = 5.355, *p* < 0.01; Mel1aR, *F*_(2,32)_ = 2.811, *p* = 0.07; Rev-erbA-α, *F*_(2,32)_ = 7.901, *p* < 0.01).

#### HPA Axis Stress-Related Genes

**FIGURE 4:**
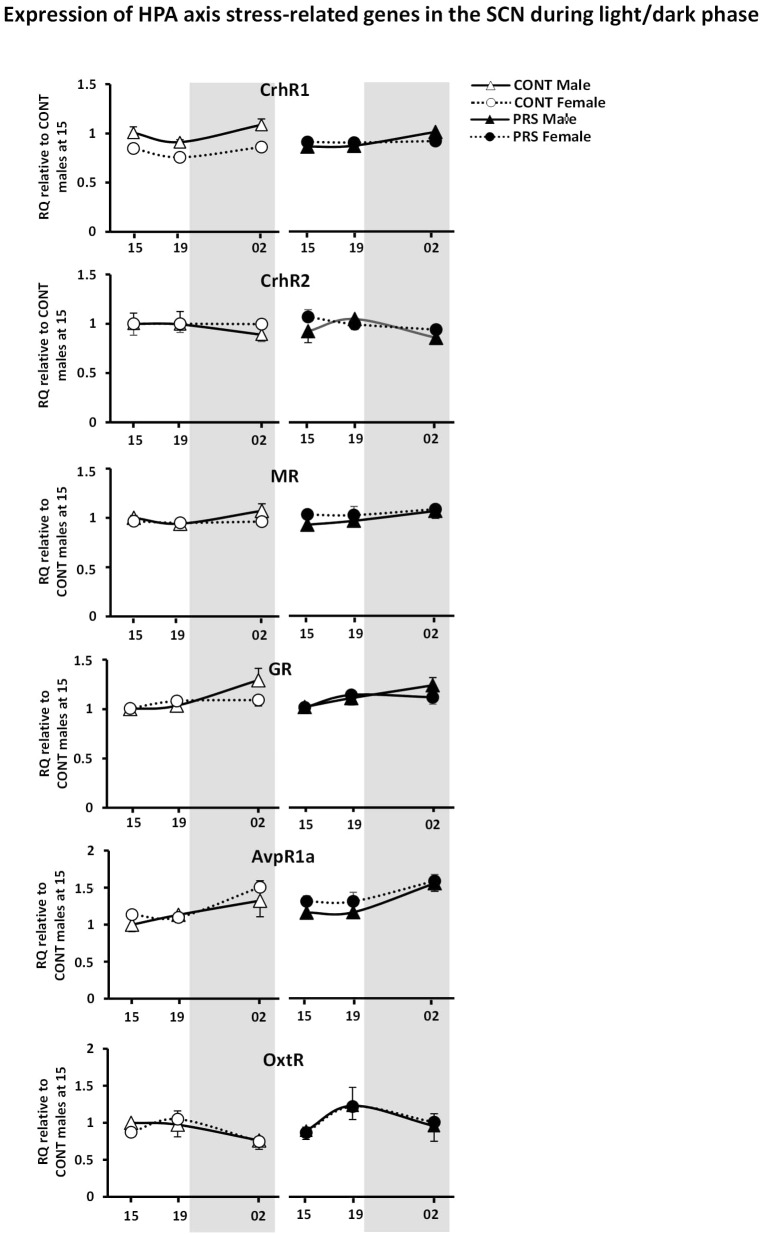
Circadian expression of HPA axis stress-related genes in the SCN of PRS and CONT animals of both sexes. Expression of selected genes in the SCN of CONT or PRS rats of both sexes was assayed by Custom Taqman qRT-PCR. Expression levels of each sample were normalized to the average levels in CONT male rats obtained at 15:00 and expressed as Relative Quantitation (RQ). All genes presented oscillation with the time exception made for *CrhR2*. PRS induced a dysmasculinization in the profile of expression for *CrhR1* in males, which showed the same expression profile as CONT females. All values are represented as mean + S.E.M of five animals per group.

The effects of PRS and sex were also observed on most of the genes involved in HPA axis activity and regulation that were included in the present study (Figure 4), such as *CrhR1, Nr3c2 (MR), AvpR1a*. Here, sex differences observed in unstressed CONT rats in the levels of *CrhR1* and *MR*, which were upregulated in males but not in females, disappeared in the PRS group (ANOVA, CrhR1 (*Group x Sex effect, F*_(1,16)_ = 18.012, *p* < 0.001, *Time effect, F*_(2,32)_ = 4.434, *p* < 0.05); MR (*Group x Sex effect, F*_(1,16)_ = 4.520, *p* < 0.05, *Time effect, F*_(2,32)_ = 3.140, p = 0.06; AvpR1a, *Sex effect, F*_(1,16)_ = 5.701, *p* < 0.05, *Time effect, F*_(2,32)_ = 11.228, *p* < 0.001). On the other hand, Nr3c1 (GR) and OxtR displayed oscillations that were independent of the main variables effect of group or sex (ANOVA, *Time effect only*: GR, *F*_(2,32)_ = 6.093, *p* < 0.01; OxtR, *F*_(2,32)_ = 2.965, *p* = 0.07) and CrhR2 expression was unchanged.

## Discussion

We showed a sex- specific profile in the unstressed group that was either exacerbated or flipped by PRS. In unstressed animals, circadian wheel running behavior was greater during the day in females and higher during the night in males. Interestingly, PRS increased and decreased total locomotor activity in males and females, respectively, and it induced a significant phase advance in the rhythm of circadian activity exclusively in males. The advance phase observed in PRS males is responsible at least in part for their hyperactivity in the dark cycle. Since in the light period PRS males behaved similarly to unstressed females, PRS induced a dysmasculinization of males’ profile. In the dark phase, PRS increased sex differences, with PRS males being hyperactive compared to PRS females, which were hypoactive with respect to all groups. The phase advance observed in PRS males is consistent with the altered circadian functioning of HPA axis activity observed in the PRS model (Koehl et al., 1999). Similar results have also been found using other models of early life stress (i.e., prenatal hypoxia, Joseph et al., 2002). The reduced locomotor activity observed in PRS females could also be related to the changes in the gonadal status that characterize the PRS model. Indeed, the reduced levels in estradiol and impaired estrous cycle observed in PRS females (Reynaert et al., 2016; Van Camp et al., 2018) is associated with the time and intensity of locomotor activity (Wollnik and Turek, 1988). Consistent with this, we have recently shown that a correction of estradiol levels in PRS females increased their rhythm of locomotor activity (Van Camp et al., 2018). The hyperactivity shown by PRS males may be related to the reduced testosterone levels observed in PRS males (Reynaert et al., 2016). In this line, in male mice, reduced testosterone levels induce a phase advance and increase levels of locomotor activity, while testosterone treatment corrects this profile (Butler et al., 2012).

Sex differences in the unstressed group were also observed in response to a chronobiological stress such as a 6-h advance phase shift, with females being more vulnerable than males since they required three more days to resynchronize to the new L/D cycle. The locomotor response to the chronobiological stress in unstressed females is consistent with the greater activation of the glucocorticoid response to stress that is generally observed in female rodents (Heck and Handa, 2018). Sex differences were observed also in PRS rats, with a greater number of days required by PRS females to re-entrain with respect to PRS males. However, we found that male and female PRS rats resynchronized their activity rhythm to the new L/D cycle more slowly than unstressed rats. Therefore, PRS males behaved like unstressed females. This specific pattern of increased time of resynchronization in the PRS animals is consistent with the increased stress corticosterone response and rhythm of secretion that has been reported in PRS animals and to a greater extent, in females (Koehl et al., 1997, 1999). Of note, significant increase in the number of days animals required in order to re-entrain to the new LD cycle following a phase shift, have been also observed in other prenatal stress paradigms in both mice and rats, following an 8-h advance/phase shift (chronic mild gestational stress, Kiryanova et al., 2017) or an abrupt 6-h delay/phase shift (prenatal hypoxia Joseph et al., 2002).

Remarkably, the pattern of locomotor activity in PRS rats under a 12:12 h L/D cycle as well as in response to the chronobiological stress was erratic and fragmented, and this was particularly evident in female PRS rats. This specific fragmentation profile has already been shown for another circadian rhythm – the sleep-wake cycle – with PRS males showing discontinuous and shorter sleep when compared to unstressed animals (Dugovic et al., 1999; Mairesse et al., 2013). Moreover, fragmented circadian activity rhythms reflect age-related alterations in the biological clock, because circadian rhythms and sleep patterns change with age (Turek et al., 1995; Van Someren, 2000). Along these lines, the characteristic pattern of fragmentation in PRS rats would lean toward an accelerated aging of the circadian clock induced by PRS. This would broaden the concept of PRS-mediated accelerated aging processes to predictive adaptation, which has already been put forward at the level of reactive adaptation. Indeed, adult PRS males display prolonged secretion of plasma corticosterone in response to stress compared to unstressed aged animals (Vallée et al., 1999), and PRS females display reduced levels of estradiol already in adulthood (Reynaert et al., 2016) similar to unstressed females during aging (Van Camp et al., 2018).

The main actor in the regulation of central and peripheral circadian rhythms is the SCN. Interestingly, we observed patterns of oscillations for the genes analyzed in the SCN that were changed by PRS and in a sex-dependent manner. Indeed, the sex-specific profile of gene expression that was reported in almost all of the genes studied in the SCN in unstressed animals, was flipped by PRS, with PRS males displaying a pattern similar to that of unstressed females. As observed in the behavioral phenotype, this pattern of dysmasculinization also affected gene expression changes.

We observed that females had downregulated expression of the genes involved in signaling regulation, with only *Dkk1* and *Igf1* being upregulated. In particular, for glucose transporters, marked sex differences exist in the gene expression profiles in rodents’ tissues (Nagai et al., 2014). Here PRS downregulated *mTor, Glut1*, and *Gsk3b* compared to unstressed males. Moreover, PRS males displayed a profile identical to unstressed females, again indicating a pattern of dysmasculinization. Down-regulation of *Glut1* in the SCN of PRS males suggests an impairment of glucose metabolism in the brain. Indeed, we previously reported downregulation of *GLUT1* in the placenta of male PRS fetuses (Mairesse et al., 2007).

Among the genes involved in clock and glucose regulation, we showed upregulation of *Clock* in unstressed males compared to unstressed females and upregulation of *Bmal, Oga*, and to a greater extent for *Mel1aR* in unstressed females. Levels of *Clock* in PRS males, were downregulated at night (02:00) with respect to unstressed males, thus displaying an unstressed female-like profile. Recent studies in rats indicate that *Clock* is expressed across brain tissues in a sex-specific pattern (Chun et al., 2015). PRS in males also downregulated *Ngf1A* during the day and higher levels during the night when compared to both unstressed animals and PRS females. Interestingly, *Bmal* and *Rev-erb-*α in PRS females displayed a masculinized profile, in that it mimicked the one observed in unstressed males. Remarkably, we observed that PRS completely flipped the sex-dependent expression of *Mel1aR* transcript levels, with PRS males showing the same profile as unstressed females with higher levels of Mel1aR expression, and PRS females displaying an expression profile similar to that of unstressed males, with reduced levels of the transcript. Within the stress response genes, we observed minor effects with upregulation of *CrhR1* mRNA levels in the SCN in males compared to females in the unstressed group. Overall, among all the genes studied, the unique profile in *Mel1aR* expression indicates that this gene is a key regulator in the programming of the circadian system induced by PRS.

## Conclusion

Our results provide a first characterization of sex differences in the behavioral and molecular chronobiological profile in unstressed rats. PRS affected males to greater extent than females on the set-up of the circadian system, and lead to the dysmasculinization of PRS males related to locomotor activity phenotype, resynchronization to the new L/D cycle, and in gene expression in the SCN by suggesting a critical role for *Mel1aR in both sexes*. To our knowledge, this is the first demonstration of sex-dependent studies on predictive adaptation programmed by early life stress. Moreover, our results provide additional information, this time at the gene expression level, on the specific pattern of dysmasculinization, which has already been reported at the sexual, physiological, and behavioral level in animal studies of prenatal stress in the past decades (Ward, 1972, 1984; Becker and Kowall, 1977; Dahlöf et al., 1977; Dörner et al., 1983; Ward and Weisz, 1984; Koehl et al., 1997, 1999; Weinstock, 2001; Reznikov and Tarasenko, 2007). The disrupting effect induced by PRS has already been shown in reactive adaptation with its effects on stress response and glutamatergic transmission in the hippocampus. The results obtained in the present study extend this disruption to the main biological clock - and therefore to predictive adaptation. Overall, perturbation of the early life environment has profound consequences on the ability of the organism to react to changes (stress response) or anticipate those changes (circadian rhythms). Therefore, it is necessary to consider sex differences in translational research, in order to gain knowledge of this under-explored field. Animal models of stress- and sleep-disorders as well as sex differences satisfy translational criteria of validity and thus provide a great opportunity of investigation.

## Ethics Statement

This study was carried out in accordance with the recommendations of the committee on animal experimentation CEEA75, Lille France. All experiments followed the rules of the European Communities Council Directive 2010/63/EU.

## Author Contributions

SM-F, JeM, GVC, JoM, FN, and SM designed the study and wrote the manuscript. SM-F, JeM, GVC, M-LR, EG, JoM, and HB conducted the experiments and analyzed the data.

## Conflict of Interest Statement

The authors declare that the research was conducted in the absence of any commercial or financial relationships that could be construed as a potential conflict of interest.

## Supplementary Material

FIGURE S1 Effect of PRS and sex differences on the expression levels of signaling- and glucose metabolism-related genes in the SCN. Expression of selected genes in the SCN of CONT or PRS rats of both sexes was assayed by Custom Taqman qRT-PCR. Expression levels of each sample were normalized to the average levels in CONT male rats obtained at 15:00 and expressed as Relative Quantitation (RQ). All genes displayed a marked effect of both sex and PRS variables or sex only. Here, gene expression is pulled over time variable. Most of the sex differences were in the direction of reduced expression levels for females, with only *Dkk1* and *Igf1* being upregulated in females. PRS males displayed reduced levels of expression for *mTor, Glut1*, and *Gsk3b* and therefore, displayed a profile identical to CONT females. Data are depicted as mean ± S.E.M of five determinations for each group (ANOVA, *Group x Sex effect*, mTor *F*_(1,16)_ = 5.750, *p* < 0.05; Glut1 *F*_(1,16)_ = 5.051, *p* < 0.05; Gsk3ß *F*_(1,16)_ = 3.288, *p* = 0.07; *Sex effect only*, mGlu2, *F*_(1,16)_ = 9.268, *p* < 0.01; mGlu3 *F*_(1,16)_ = 5.894, *p* < 0.05; Akt1, *F*_(1,16)_ = 9.747, *p* < 0.01; Disc, *F*_(1,16)_ = 5.991 *p* < 0.05; Dkk1, *F*_(1,16)_ = 4.855 *p* < 0.05; Glast, *F*_(1,16)_ = 7.617, *p* < 0.01; InsR, *F*_(1,16)_ = 3.893, *p* = 0.06; *Time effect*, mTor *F*_(2,32)_ = 4.180, *p* < 0.05; Glut1, *F*_(2,32)_ = 6.046, *p* < 0.01; Akt1, *F*_(2,32)_ = 5.151, *p* < 0.05; Disc1, *F*_(2,32)_ = 8.906, *p* < 0.001; InsR, *F*_(2,32)_ = 4.050, *p* < 0.05). ^∗^*p* < 0.05 PRS vs. CONT within the same sex group; ^#^*p* < 0.05 and ^##^*p* < 0.01 females vs. males within the CONT or PRS group.
